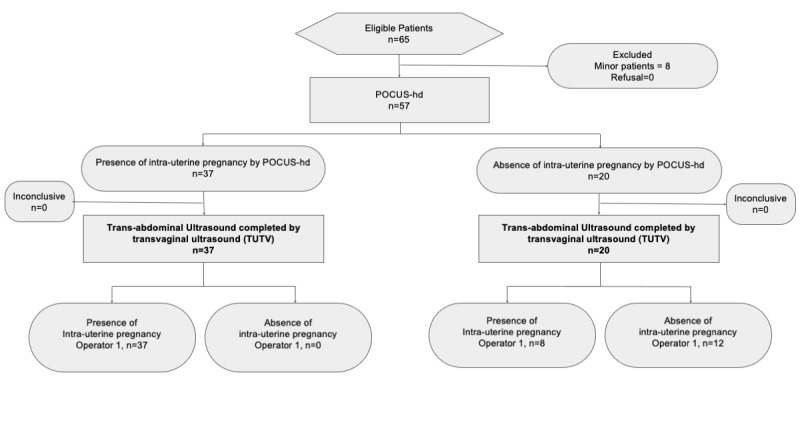# Intrauterine Pregnancy Detection and Gestational Age Assessment During Early Pregnancy by a Handheld Point-Of-Care Ultrasound Device Compared to a High-End Ultrasound System. An Accuracy and Reliability Study

**DOI:** 10.24908/pocus.v7i2.15458

**Published:** 2022-11-21

**Authors:** Mariela Skendi, Roxane Liard, Charlotte Besacier, Jean-Michel Correas, Sohela Moussaoui, Julie Chastang, Gladys Ibanez

**Affiliations:** 1 Department of General Practice, Faculty of Medicine, Sorbonne University Paris France; 2 Department of Adult Radiology, Necker Hospital Paris France; 3 Institut Pierre Louis d'Epidémiologie et de Santé Publique (IPLESP) Paris France

**Keywords:** Accuracy, Reliability, Pregnancy, Gestational age, Point-of-care ultrasound

## Abstract

**Objective:** The main objective of this study is the evaluation of the accuracy and reliability of a handheld point of care ultrasound device (POCUS-hd) for intrauterine pregnancy (IUP) detection compared to comprehensive reference transabdominal ultrasound (TU). The secondary objectives were to evaluate POCUS-hd for intrauterine pregnancy (IUP) detection compared to transabdominal and transvaginal ultrasound (TUTV), evaluate the inter-device agreement and inter-rater reliability of gestational age during early pregnancy. **Methods: **It is an observational transverse study with consecutive patient recruitment. Two blinded operators systematically used POCUS-hd and reference transabdominal ultrasound for IUP diagnosis. The accuracy of POCUS-hd for IUP diagnosis was expressed as sensitivity, specificity, negative predictive value (NPV) and positive predictive value (PPV). The gestational age (GA) was assessed based on the crown-rump length. The reliability and agreement of gestational age evaluation were assessed by Bland-Altman plots, kappa statistic, and intraclass correlation coefficients (ICC). **Results: **POCUS-hd compared to TU had a sensitivity of 95-100%, a specifcity of 90-100%, PPV of 95-100% and NPV of 90-100%. Inter-rater agreement for IUP detection using POCUS-hd was very good, kappa=1.0; CI95% [0.9-1.0]. The inter-device agreement limits (mean difference ± 2SD) for GA were: -3 to +2.3 days by Operator 1, -3.4 to +3.3 days by Operator 2 for POCUS-hd vs. TU and -3.1 to +2.3 days for POCUS-hd versus TUTV. **Conclusion:** This handheld POCUS device is an accurate and reliable diagnostic tool that can be used for IUP positive findings and GA assessment during early pregnancy by clinicians in family planning settings or general practice.

## Background

The past decade saw the development and increased popularity of new point of care ultrasound (POCUS) devices. These portable devices have very fast start times, when compared to conventional ultrasound machines, and enable clinicians to perform POCUS at the bedside in clinical units. The further miniaturization of the machines gave birth to a new concept - “echoscopy” – defined in 2013 by the European Federation of Societies for Ultrasound in Medicine and Biology (EFSUMB)^1^, ^2^ as part of three levels of ultrasound: echoscopy, POCUS and comprehensive ultrasound. While POCUS highlights the setting where the ultrasound exam is performed, echoscopy is defined by its ability to answer a simple targeted clinical question asked by the clinician at the bedside that can be documented in the patient’s chart and does not require a detailed imaging report. It is the intention of the clinician and the need to answer a specific clinical question that defines the type of ultrasound performed. Should the physician wish to perform a detailed exam to explore a region of organs, sophisticated devices used for comprehensive ultrasound machines are better adapted. Echoscopy and POCUS can be performed with handheld devices while comprehensive ultrasound with more sophisticated equipment 2.

For patients presenting lower abdominal pain and/or vaginal hemorrhage in early pregnancy, it is important for the clinician both to confirm the presence of an intrauterine pregnancy (IUP) as well as estimate the gestational age (GA), for clinical decision making later in pregnancy or voluntary pregnancy termination 3. Before using these new miniaturized ultrasound devices to answer such clinical questions in everyday practice, it is important to evaluate their accuracy and reliability compared to a high-end system.

The principal objective was the evaluation of the accuracy and reliability of handheld point of care ultrasound device (POCUS-hd) for IUP detection compared to comprehensive reference transabdominal ultrasound (TU). The secondary objectives were to evaluate POCUS-hd for IUP detection compared to transabdominal and transvaginal ultrasound (TUTV), evaluate the inter-device agreement and inter-rater reliability in calculating GA in the first trimester of pregnancy.

## Methods

### Study Design

This was an observational transverse monocentric study conducted according to the STARD and GRRAS guidelines for accuracy and reliability 4, 5. All studies were performed at the Family Planning Clinic at the Cochin Port-Royal University Hospital in Paris, France. 

The first part of the study compared the accuracy of a POCUS-hd compared to TU and TUTV for the detection of IUP and the inter-operator agreement for IUP detection. 

The second partof the study evaluated the inter-device agreement on GA measurement. The GA obtained using POCUS-hd was compared to the TU measurement. The inter-rater variability for GA measurement was then calculated for each operator. Two blinded operators scanned independently the same population of patients in alternating order, on the same day at 5-10 minutes intervals. Operator 1 performed POCUS followed by TU for all patients and a TUTV for all pregnancies younger than 6 weeks of gestation or whenever the embryo could not be visualized transabdominally according to the usual practice at the clinic. Both POCUS and the reference comprehensive ultrasound were performed on the same day during the patient’s visit to the family planning clinic.

Operator 2 performed POCUS-hd followed only by a TU. At the end of each study, both operators would fill a written report and would communicate the results of their respective scan to the patient. Patients had no specific preparation for the study, like fasting or full bladder requirement. 

### IUP definition and GA calculation

The presence of an IUP was confirmed by the visualization of the double decidual sac sign on B-mode with either an embryo or a yolk sac.

The GA was assessed based on the crown-rump length (CRL) using the following equation 6: gestational age (days) = 8.052*(1.037*CRL)^1/2^+23.73.

The mean diameter of the gestational sac was not used for GA calculation due to its higher variability and less precise GA estimation 7, 8, 9, 10, 11, 6, 12. Basic settings were used such as gain, depth, zoom, and use of calipers for measurements of CRL.

### Population

We aimed to recruit 65 consecutive patients who visited the Family Planning Clinic at the Cochin Port-Royal University Hospital between May and July 2016. Among this population, pregnancy was either confirmed or suspected. Patients would come in with a positive urinary pregnancy test, a positive plasmatic beta human chorionic gonadotropin (hCG), a delay in the onset of menses, abdominal pain with/or vaginal bleeding and for a follow-up visit to confirm pregnancy termination. Patients were included if they were at least 18 years of age. Patients were excluded from the study if they were under 18 years, had twin gestations, refused to sign a consent form or if the image acquisition was incomplete due to technical difficulties with the ultrasound machine. 

### Operators

Operator 1 was a general practitioner (GP) who had been working and using ultrasound at the family planning clinic for 5 years. Operator 2 was a GP who had a general 2-year ultrasound diploma and had finished a 6-month training at the family planning clinic.

### Ultrasound Device

POCUS was performed with a handheld Visiq Philips device that weighed 1 kg, had an average start time of 30 seconds and was connected to a C5-2°MHz transducer through USB port. The comprehensive reference ultrasound was performed on the ProSound Alpha 6 machine using UST-9123 6-2 MHz and UST-9124 7.5-3 MHz transducers. 

### Data Storage and Interpretation

**Figure 1:**
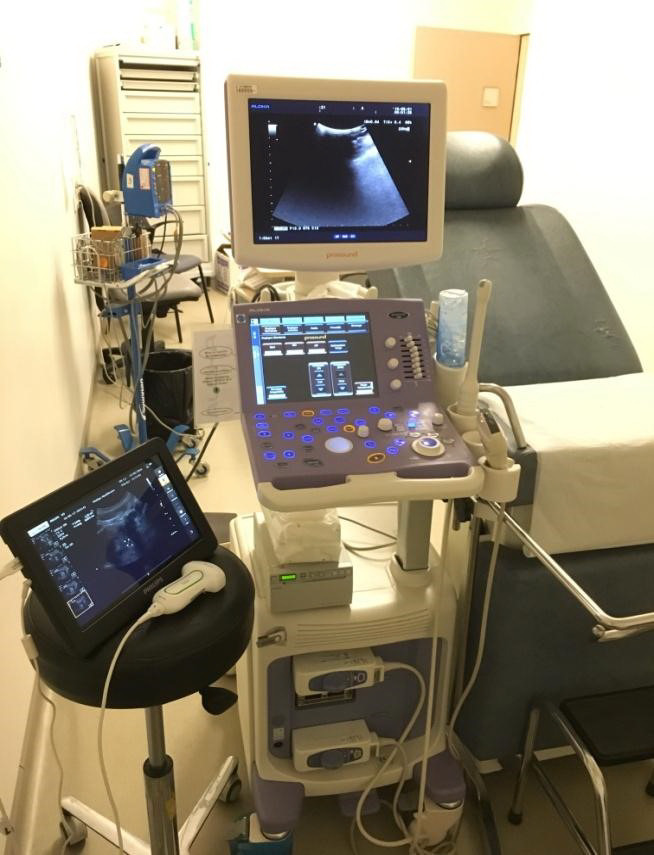
POCUS handheld device (on the left) and comprehensive ultrasound device (on the right) used for IUP detection and gestational age measurement.

Images obtained by POCUS-hd were stored as DICOM files on the Visiq Philips ultrasound device. Images obtained by the transabdominal ultrasound machine were stored on the ProSound Alpha 6 machine. Both operators recorded their findings and image interpretations on paper files (Figure 1)**.**

### Statistical Analysis

The accuracy of POCUS-hd was calculated in terms of sensitivity, specificity, negative predictive value and positive predictive value using contingency tables for Operators 1 and 2. Inter-rater agreement on IUP detection was evaluated by the kappa statistic. Inter-device agreement for GA evaluation was calculated using Bland-Altman plots. Inter-rater variability for GA measurement with POCUS-hd was calculated using the intraclass correlation coefficient (ICC) and Bland-Altman plots 12. Data analysis was performed by the Department of General Practice at Sorbonne University, using Stata and R Studio software.

## Results

**Figure 2:**
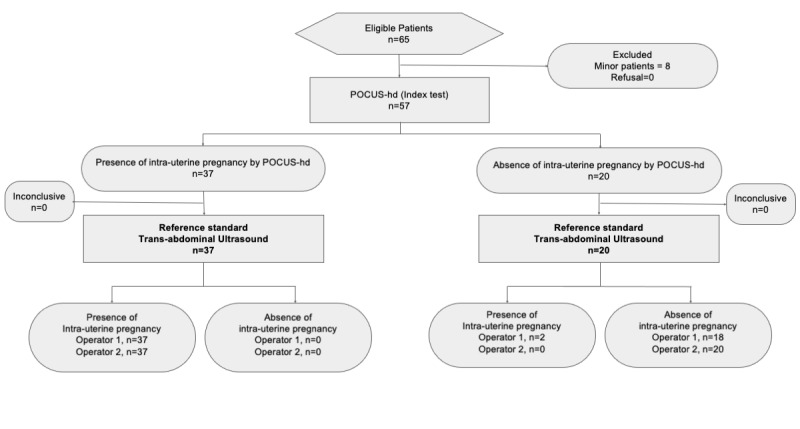
Flow diagram of POCUS-hd accuracy where the reference is transabdominal ultrasound performed by Operator 1 and Operator 2.

Among the 65 eligible women, 57 were enrolled in the study (Table 1). On standard transabdominal ultrasound, there were 37 IUPs detected, among whom 34 had visible embryos, 3 had gestational sacs with yolk sacs according to POCUS-hd. On TUTV there were 45 IUP detected, among whom 41 had visible embryos, 4 had gestational sacs with yolk sacs according to TUTV (Figure 2, Figure S1 suppl). 

**Table 1:** Patient characteristics (n=57).

**Patients**	**m ± sd**
Age (years)	27.3 ± 6
Gestational age (days)	50.9 ± 14
Weight (kg)	63.6 ± 13
Height (cm)	165.7 ± 6
Body Mass Index (kg/m²)	23.2 ± 5

### Accuracy

POCUS-hd accuracy was calculated through contingency tables. The sensitivity of POCUS-hd for IUP detection was 95-100% (35/37 for Operator 1 and 37/37 for Operator 2) when compared to TU alone. The specificity for POCUS-hd for IUP detection was 90-100% (18/20 for Operator 1 and 20/20 for Operator 2) when compared to TU alone. The positive predictive value (PPV) was 95-100% (35/37 Operator 1, 37/37 Operator 2). The negative predictive value (NPV) was 90-100% (18/20 Operator 1, 20/20 Operator 2).

The sensitivity of POCUS-hd for IUP detection was 82% (37/45 by Operator 1) when compared to TUTV. The specificity for POCUS-hd for IUP detection was 100% (20/20 by Operator 1) when compared to TUTV. The PPV was 100% (37/37) and NPV was 60% (12/20) where the reference ultrasound was TUTV. 

Inter-rater agreement for IUP detection by POCUS-hd was excellent, kappa=1.0; CI_95% _[0.9-1.0] (Table 2). 

**Table 2:** Diagnostic accuracy ofechoscopy for intrauterine pregnancy detection compared to comprehensive ultrasound (n=57).

		**Sensitivity**	**Specificity**	**Positive** **Predictive Value**	**Negative** **Predictive Value**
**Transabdominal** **Ultrasound (TU)**	**Operator 1**	95%	100%	95%	90%
	**Operator 2**	100%	100%	100%	100%
**Transabdominal and Transvaginal Ultrasound (TUTV)**	**Operator 1**	82.3%	100%	100%	60%

### Reliability

**Figure 3:**
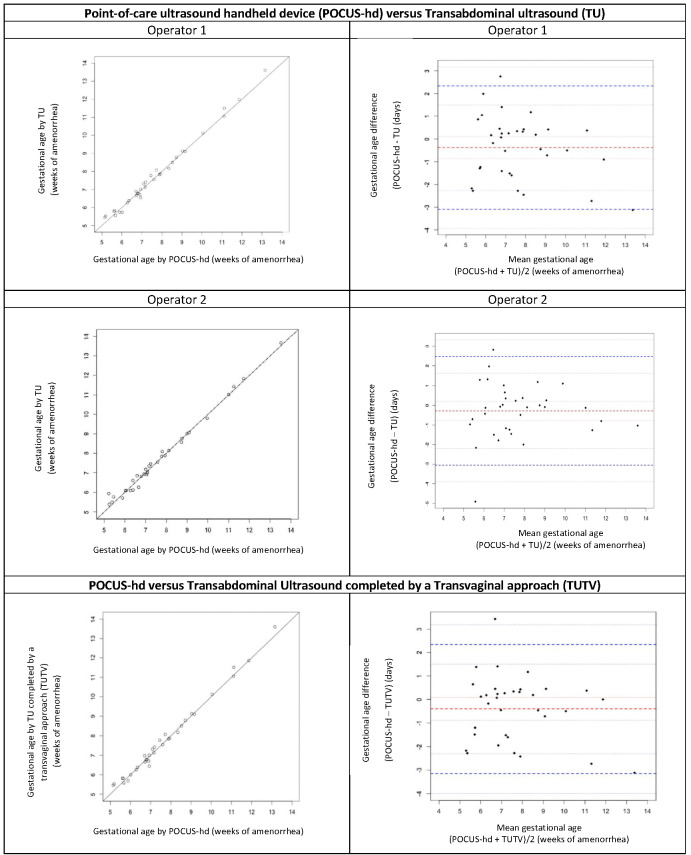
Plot of gestational age measured by POCUS-hd versus transabdominal reference ultrasound (TU), by Operator 1 (top left), Operator 2 (middle left). Plot of gestational age measured by POCUS-hd versus transabdominal ultrasound completed by transvaginal ultrasound (TUTV), by Operator 1 (bottom left). Bland-Altman plot comparing gestational age measurement by point-of-care ultrasound handheld device (POCUS-hd) versus Transabdominal ultrasound (TU) by Operator 1 (top right), Operator 2 (middle right). Bland-Altman plot comparing gestational age measurement by point-of-care ultrasound handheld device (POCUS-hd) versus Transabdominal ultrasound completed by transvaginal approach (TUTV) by Operator 1 (bottom right).

Agreement limits of POCUS-hd vs. TU (mean difference ± 2SD) were -3.0 to +2.3 days for Operator 1 and -3.4 to +3.3 days for Operator 2. Agreement limits of POCUS-hd vs. TUTV were -3.1 to +2.3 days. The inter-device agreement (POCUS-hd vs. TU and POCUS-hd vs. TUTV) for GA estimation was very good (Figure 3).

**Figure 4:**
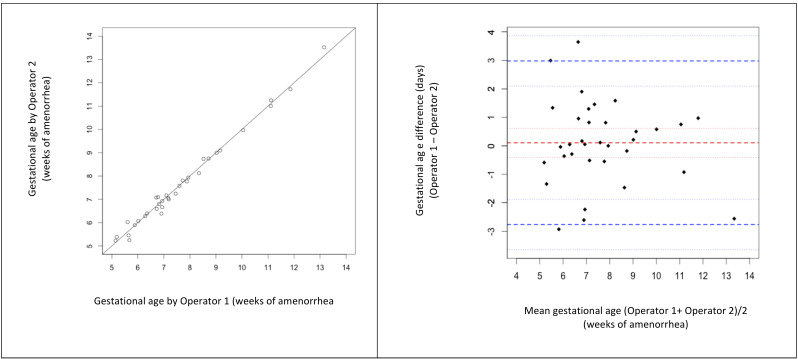
Inter-rater variability for gestational age measurement by POCUS-hd.

The inter-rater agreement of GA by POCUS-hd was excellent, ICC = 0.99, CI 95% [0.98 - 0.99] and agreement limits on the Bland-Altman plot were -2.7 to +3 days (Figure 4).

## Discussion

The handheld POCUS device, compared to transabdominal ultrasound and transabdominal ultrasound completed with transvaginal, was highly accurate for IUP detection and GA assessment. The study showed excellent agreement of POCUS-hd versus TU and POCUS-hd versus TUTV for GA measurement as agreement limits of POCUS-hd [+/-3 days] are within the precision limits of ultrasound dating of [+/-5 days] days used in clinical practice 7, 13. The reproducibility of gestational age measurements by POCUS-hd between the 2 operators was very good.

POCUS-hd is intended to be used by clinicians who need to determine the location of a pregnancy in the first trimester at the bedside during the clinical examination. They may then, depending on their abilities, date the pregnancy if needed 8. In the event of a negative result where intrauterine pregnancy cannot be confirmed, it is up to the clinician to decide whether to continue the investigations and within what time frame to repeat POCUS, or request a comprehensive ultrasound. Of the 65 patients recruited for this study, 8 were excluded because they were under 18 years of age. The remaining 57 patients, all consented to be part of the study.

To this day, only a few studies have evaluated the accuracy of a handheld ultrasound device for routine obstetrical examination during early pregnancy but there is no study that evaluates their accuracy and reliability in a context of pregnancy termination. A systematic review published in 2019 by Rykkje et al. comparing hand-held ultrasound devices with high-end ultrasound showed a good overall agreement for obstetrics and gynecology use. The results of our study with a Visiq Philips handheld device are comparable to the results of 3 obstetrics/gynecology studies during the first semester at an emergency setting in terms of reliability where a Vscan was used 14. One of the strengths of this study is to evaluate another handheld device, Visiq/Philips, in a context of pregnancy termination in a family planning clinic.

Sayasneh et al. evaluated the validity of a POCUS-hd device, Vscan, in a population of 101 patients with signs of pelvic pain or hemorrhage during their first trimester. There was “good” to “very good” concordance between the Vscan and the transabdominal and transvaginal ultrasound for the detection of an embryo, a gestational sac, cardiac activity, with kappa coefficients of 0.844, 0.843 and 0.729, respectively (p <0.0001). The concordance for CRL and mean diameter of the gestational sac measurement was very good with an ICC > 0.9 (p <0.0001) 15. These results are in agreement with the results of our study covering 37 women which found a good concordance between POCUS-hd and the transabdominal ultrasound to measure gestational age based on crown-rump length, ICC = 1.0 (p <0.0001).

Several studies on the different fields of application of POCUS suggest that its greatest potential and impact on morbidity and mortality is in obstetrics. A study on the use of POCUS by midwives in Zambia, for example, showed that they can be trained to perform POCUS, answer simple obstetric clinical questions, and impact clinical decision-making 16, 17, 18, 19.

One of the limitations of this study is that it does not assess intra-operator variability. This possibility was discussed during the design of the study but additional measures would have extended the duration of the examination and might have become uncomfortable for patients. For this reason and for patients’ comfort, transvaginal ultrasound was not repeated by Operator 2 but was rather performed only once by Operator 1 as part of the usual practice. 

Among obstetric studies, bedside ultrasound is easily accepted and allows accurate monitoring of pregnancy after 5 weeks of gestation. Pelvic pain in early pregnancy may be secondary to an ectopic pregnancy in the absence of a uterine gestational sac and the presence of an adnexal mass or intraperitoneal free fluid. The results of a meta-analysis on the diagnosis of ectopic pregnancy by bedside ultrasound performed by emergency physicians show a high specificity and high sensitivity in the localization of a pregnancy but remain operator-dependent 17, 20, 21, 22, 23.

The results of our study showed that the diagnostic performance of POCUS-hd to detect intrauterine pregnancy was satisfactory and could be used in the family planning clinic. The reliability of a handheld POCUS-hd device to evaluate GA during the first trimester was comparable to conventional ultrasound with an accuracy of +/- 3 days. The population in this study included adult women in the first trimester of their pregnancy. However, the study may be extended to other populations. POCUS-hd can also be used in other clinical situations such as confirming the proper positioning of an intrauterine device. In the context of gynecological emergencies, it can assess the viability of the pregnancy. POCUS-hd can also be used to diagnose other (non-obstetric) pathologies such as a pelvic mass or intraperitoneal free fluid. Its usefulness in cardiac, renal, vascular, hepatosplenic and musculoskeletal pathologies has been well-established.

As technology progresses, both image resolution and POCUS-hd affordability will undoubtedly improve. In the near future, physicians and medical students will be equipped more easily and educators will teach ultrasound skills during medical school or during continuing medical education activities.

## Conclusions

This handheld POCUS device seems to be an accurate and reliable diagnostic tool that can be used for IUP detection and GA assessment during early pregnancy by clinicians in the family planning setting or general practice. These portable devices enable clinicians to perform POCUS at the bedside in clinical units, help improve the accuracy of the physical exam and improve patient care.

## Ethics statement and consent

Patients were informed before the study orally and in writing. All patients signed a written consent form before participating in the study.In conformity with  the French regulation, authorizations for the study were obtained by the institution of Advisory Committee on the Processing of Research Information (CCTIRS) and National Data Protection Commission (CNIL) #2070527. Considering the study did not change the usual practice and it did not involve any risk for the patients, a formal ethical approval was not required. Patients or the public were not involved in the design, or conduct, or reporting, or dissemination plans of this research.

## Funding

The Visiq ultrasound equipment was supplied by Philips on a temporary loan for the study duration, at the request of the author (MS). Philips had no role in study design, data collection, analysis, decision to publish, or preparation of the manuscript.

## Supplementary Material

Supplementary Figure S1Flow diagram of POCUS-hd accuracy where the reference is transabdominal ultrasound completed by transvaginal ultrasound performed by Operator 1.